# Asymptomatic carotid stenosis is associated with both edge and network reconfigurations identified by single-subject cortical thickness networks

**DOI:** 10.3389/fnagi.2022.1091829

**Published:** 2023-01-12

**Authors:** Jinxia Ren, Dan Xu, Hao Mei, Xiaoli Zhong, Minhua Yu, Jiaojiao Ma, Chenhong Fan, Jinfeng Lv, Yaqiong Xiao, Lei Gao, Haibo Xu

**Affiliations:** ^1^Department of Radiology, Zhongnan Hospital of Wuhan University, Wuhan, Hubei, China; ^2^Department of Nuclear Medicine, Zhongnan Hospital of Wuhan University, Wuhan, Hubei, China; ^3^Center for Language and Brain, Shenzhen Institute of Neuroscience, Shenzhen, China

**Keywords:** cortical thickness, structural covariance, brain network, vascular cognitive impairment, graph theory

## Abstract

**Background and purpose:**

Patients with asymptomatic carotid stenosis, even without stroke, are at high risk for cognitive impairment, and the neuroanatomical basis remains unclear. Using a novel edge-centric structural connectivity (eSC) analysis from individualized single-subject cortical thickness networks, we aimed to examine eSC and network measures in severe (> 70%) asymptomatic carotid stenosis (SACS).

**Methods:**

Twenty-four SACS patients and 24 demographically- and comorbidities-matched controls were included, and structural MRI and multidomain cognitive data were acquired. Individual eSC was estimated via the Manhattan distances of pairwise cortical thickness histograms.

**Results:**

In the eSC analysis, SACS patients showed longer interhemispheric but shorter intrahemispheric Manhattan distances seeding from left lateral temporal regions; in network analysis the SACS patients had a decreased system segregation paralleling with white matter hyperintensity burden and recall memory. Further network-based statistic analysis identified several eSC and subgraph features centred around the Perisylvian regions that predicted silent lesion load and cognitive tests.

**Conclusion:**

We conclude that SACS exhibits abnormal eSC and a less-optimized trade-off between physical cost and network segregation, providing a reference and perspective for identifying high-risk individuals.

## 1. Introduction

Clinically “asymptomatic” carotid stenosis, even without stroke, is associated with cognitive impairment (de Weerd et al., 2014; Lal et al., 2017; Lazar et al., 2021), which is generally characterized by reduced processing speed and learning/memory capacity (Martinić-Popović et al., 2009; Lazar et al., 2021). As such, cognitive status has been suggested as an indicator of disease progression and efficacy monitoring in these patients (Lattanzi et al., 2018). However, the neuroanatomical basis underlying cognitive impairment remains largely unclear.

Neuroimaging studies have revealed notable structural alterations in asymptomatic carotid stenosis. Morphological analyses, for example, reveal asymmetrical (Gao et al., 2021b) and stenosis-ipsilateral dominated gray matter (GM) atrophies (Avelar et al., 2015), cortical thinning in the *Perisylvian* regions (Gao et al., 2021a), and accelerated aging changes (Alhusaini et al., 2018). Lesion mapping has reported prominent subcortical microinfarcts and white matter hyperintensity (WMH; Kandiah et al., 2014; van Veluw et al., 2017). Structural imaging also identifies reduced white matter integrity and fiber density in such patients (Cheng et al., 1970; Meng et al., 2017; Gao et al., 2019). Moreover, these patients are reported to show compromised hemodynamics (Silvestrini et al., 2009; Balucani et al., 2012), glymphatic dysfunction and enlarged perivascular spaces (Liu et al., 2021). These lines of evidence suggest that extensive morphological changes have occurred in these patients, and morphometric network analysis based upon may provide rich information for understanding the neuroanatomical basis of cognitive impairment and predicting long-term risks.

A multivariate methodology is the structural covariance network analysis (Alexander-Bloch et al., 2013), which assumes that morphological measures [e.g., cortical thickness (CT)] of one brain region and another structurally and functionally connected one co-vary, forming different communities. These co-variations are underpinned by gene co-expression (Romero-Garcia et al., 2018), systematically change across lifespan development (Montembeault et al., 2012; Kuo et al., 2020), and are sensitive to the early stages of neuropsychiatric diseases (e.g., Coppen et al., 2016; Liu et al., 2019; Qing et al., 2021). For example, normal aging is characterized by a decrease in GM covariance connectivity in higher-order associative regions, especially those involved in the semantic, executive control, and default mode functions (Montembeault et al., 2012). However, most previous studies of morphometric networks are based on group-level covariance across subjects. While this analysis can reveal brain network architecture, it cannot derive individual-level network measures, which greatly reduces its clinical utility for individual patients.

Consequently, individualized single-subject morphometric network analyses are emerging as a new area of research (Tijms et al., 2012; Li et al., 2017; Xue et al., 2022). They can provide measures similar to those derived from functional connectivity with functional MRI or structural connectivity with diffusion MRI tractography, holding promise for clinical settings. The construction of individual morphometric network is generally by estimating the interregional/areal relationships, e.g., probability distributions, distances, and similarities, of morphological features (e.g., GM volume, CT, cortical gyrification, sulcus depth, and cortical complexity; Tijms et al., 2012; Wang et al., 2016; Li et al., 2017; Raamana and Strother, 2018). The pairwise estimates of morphological features are termed edges in network analysis. The edges and their inter-edge relationships are called edge-centered brain connectivity, which is a new direction in human brain connectomics.

Asymptomatic carotid stenosis is a condition characterized by potential chronic hypoperfusion, microembolism, and hemodynamic burdens caused by internal carotid stenosis (Cheng et al., 1970; Marshall et al., 2017; Wang et al., 2017). This condition primarily affects the *Perisylvian* cortical reorganization and topographically involves somatosensory/motor, semantic, silent processing, and frontoparietal systems, as well as cognitive functions such as processing speed and semantic memory (Avelar et al., 2015; Gao et al., 2019; Nickel et al., 2019; Gao et al., 2021b). Thus, an investigation of the morphometric network may provide us with a better understanding of the neuroanatomical basis of cognitive impairment in carotid stenosis. Network edges derived from interareal histogram weights form rich connectivity information, and promote the identification of the edges that closely relate to cognitive impairment (Raamana et al., 2015; Wang et al., 2016, 2018; Raamana and Strother, 2020; Li et al., 2021; Peng et al., 2022). However, it is unknown to what extent asymptomatic carotid stenosis may affect the edges and network architecture that are closely associated with cognitive impairment and serve as potential imaging markers.

In this study, we used a novel individual approach to construct single-subject CT networks and contrasted unilateral (>70%) severe asymptomatic carotid stenosis (SACS) patients with demographically and comorbidities-matched healthy controls (HC). We estimated edge-centric structural connectivity (eSC) derived from the pairwise Manhattan distances of regional CT histograms. We expected that the SACS showed abnormal eSC in the *Perisylvian* and language-related regions and that this eSC was associated with cognitive impairment in SACS patients. Furthermore, large-scale structural networks reconstructed from the eSC would exhibit lower network segregation and efficiency globally.

## 2. Materials and methods

### 2.1. Participants

This study included 24 SACS patients and 24 demographically- and comorbidities-matched elder HC. Patients were 55–80 years; unilateral internal carotid artery (ICA) stenosis ≥70% and contralateral ICA stenosis <50%; free of stroke, transient ischemic attack, or dementia; Modified Rankin Scale (Sulter et al., 1999) score of 0 or 1. Patients would be excluded if they had posterior circulation diseases, Mini-Mental State Examination (MMSE; Tombaugh and McIntyre, 1992) score < 26, modified Rankin scale (Sulter et al., 1999) ≥ 2 (functional disability), severe systemic/neuropsychiatric diseases, education <6 years, or contraindications for MRI. Detailed demographic and participant information can be found in our recent publications (Gao et al., 2019, 2021b). The present study was approved by the Medical Ethics Committee of Zhongnan Hospital of Wuhan University, and all participants gave written informed consent.

### 2.2. Neurobehavioral assessments

Neurobehavioral batteries were administered to the participants and focused on different cognitive domains: (i) global cognition, including the MMSE and the Montreal - Cognitive Assessment (MoCA; Nasreddine et al., 2005); (ii) executive functions, including the Digit Symbol Tests (DST); and (iii) memory, including the Rey Auditory Verbal Learning Tests. A detailed description of these neurocognitive tests can be found elsewhere (Gao et al., 2019, 2021b).

### 2.3. MRI data acquisition

MRI data were acquired on a Siemens 3.0 T scanner (MAGNETOM Trio, Germany), including 3D T1-weighted anatomical images [repetition time (TR)/echo time (TE)/inversion time (TI) = 2250/2.26/900 ms, slice thickness = 1 mm, flip angle (FA) = 9°, no interslice gap, 176 sagittal slices, matrix size = 256 × 256], and T2-weighted fluid-attenuated inversion recovery (FLAIR) images (TR/TE/TI = 6,000/388/2200 ms, FA = 120°, slice thickness = 1 mm, no interslice gap, voxel size = 0.5 mm × 0.5 mm × 1 mm, 160 axial slices) covering the whole brain. Other sequences not included in this study were not described here.

### 2.4. Structural image preprocessing

T1 anatomical images were pre-processed using FreeSurfer’s (version 6.0)^1^ “recon-all” pipeline. Individual CT was generated and quality assurance procedures were carried out. The CT surfaces were spatially smoothed with a 15-mm full width at half maximum (FWMH) Gaussian kernel, as recommended by (Li et al. (2021)), and were verified using a 25-mm FWMH Gaussian kernel.

### 2.5. Edge-centric structural connectivity estimated from pairwise cortical thickness histograms

**Figure 1:**
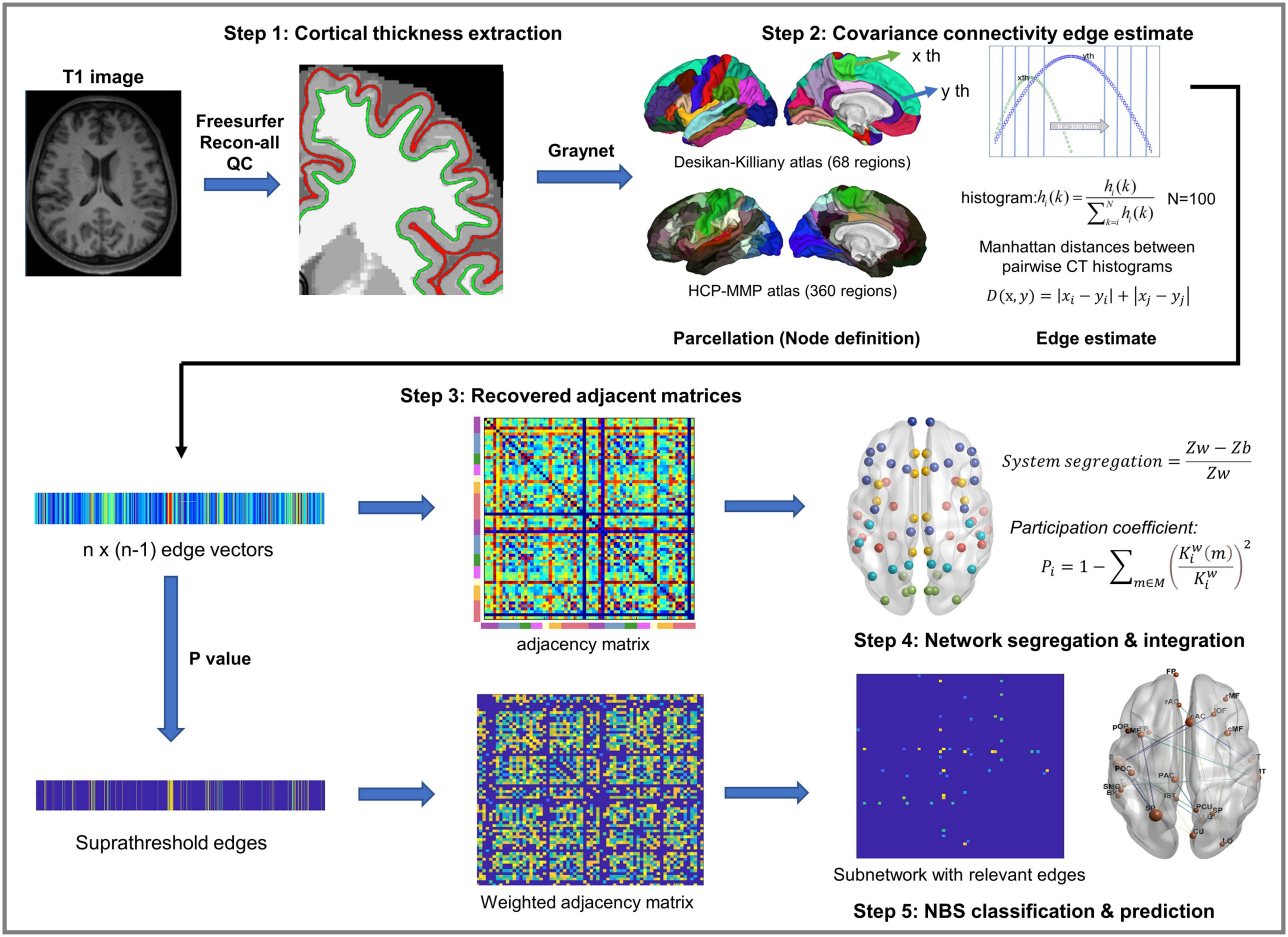
Schematic overview. Step 1: T1 anatomical image reconstruction and cortical thickness extraction. Step 2: Single-subject cortical thickness connectivity was constructed using the Graynet software. Step 3: Connectivity edges were computed as vectors, and then recovered into adjacent matrices for group statistics. Step 4: Network segregation and integration calculation. Step 5: Suprathreshold edges were used for further network-based statistics classification and regression.

Graynet^2^ (Raamana and Strother, 2018) was used to construct individualized single-subject CT networks. Network nodes were defined using the two atlases, namely the Desikan-Killiany atlas (Desikan et al., 2006) and the Human Connectome Project (HCP) MultiModal Parcellation (HCP-MMP) atlas (Glasser et al., 2016). They consist of 68 (34 for each hemisphere) and 360 areas (180 for each hemisphere), respectively (Figure 1).

For each parcellation/node, 5% outliers from the distribution of CT values were discarded to improve the robustness of the feature. The residual distribution was transformed into a histogram by binning it into N = 100 equally spaced bins. The histogram counts were then normalized by for k = 1: N, where *hi* is the node *i* histogram (Figure 1, step 2). This approach computes the pairwise edge-weight for the two nodes *i* and *j*, regardless of the number of vertices in the two nodes (Figure 1, step 2). For a detailed method description, please see the original articles (Raamana et al., 2015; Raamana and Strother, 2020). Edges (i.e., eSC) were defined as the pairwise Manhattan distance weights of interareal CT histograms. A shorter Manhattan distance reflects a stronger morphological “connection,” whereas a longer one reflects a longer traveling distance or a weaker connection. Finally, each eSC was rescaled to [0, 1] using the Min-Max Scaling (Figure 1).

### 2.6. Network segregation and integration

The rescaled matrix was further subtraction normalized (i.e., each edge was subtracted by 1) and the diagonal values were set to zeros. This transformation generates an adjacent matrix that reflects eSC strength, which is consistent with a traditional SC matrix.

#### 2.6.1. Within-system and between-system connectivity

Since morphological networks, especially the network derived from morphological CT connectivity reported here, have an apparent distinct modular or community structure from those derived from functional connectivity MRI. Thus, a direct use of the community index as defined in Chan et al. (2014) can be problematic. To reconcile this issue, we used lobar level parcellations as initially defined in the Desikan-Killiany atlas to assign the whole network nodes into five systems (Desikan et al., 2006), i.e., frontal, parietal, temporal, occipital, and cingulate cortices. Network segregation and integration were therefore computed on this basis. Within-system connectivity was calculated as the mean eSC of all nodes of that system to each other (i.e., the mean eSC between all frontal nodes to all other frontal nodes). Conversely, between-system connectivity was calculated as the mean eSC between each node of a system and all nodes of all other systems (i.e., the mean eSC between all frontal nodes and all other nodes in the cortex).

#### 2.6.2. System segregation

The system segregation was computed as values of within-system connectivity in relation to between-system connectivity (Chan et al., 2014), as follows:


$$\mathrm{System segregation}=\frac{{\bar{Z}}_{w}-{\bar{Z}}_{b}}{{\bar{Z}}_{w}}$$

Where ${\bar{Z}}_{w}$ is the mean eSC between nodes within the same system and ${\bar{Z}}_{b}$ is the mean eSC between nodes of one system to all nodes in other systems.

#### 2.6.3. System integration

The system integration was computed using the participation coefficient, which measures to what degree a node diversely connects between systems, that is, strong cross-regional information integration capabilities (Rubinov and Sporns, 2010), as follows:


$$\mathrm{Participation coefficient}=1-\underset{m\in M}{\sum }{\left(\frac{K_{i}^{w}\left(m\right)}{K_{i}^{w}}\right)}^{2}$$

Where $K_{i}^{w}\left(m\right)$ is the weighted connections of node i with nodes in system m (a system to which node i does not belong) $K_{i}^{w}$ is the total weighted connections node i exhibits. Higher participation coefficient values indicate proportionally greater communication with nodes in other systems.

### 2.7. Network-based statistic prediction

To validate the case–control classification ability of the eSC measures, we adopted a recently improved and validated method called network-based statistical (NBS) prediction (Serin et al., 2021) to overcome the small sample size of this study.

The NBS-Predict toolbox^3^ was used to detect and identify SACS-related abnormal eSC with both hyperconnectivity and hypoconnectivity. Parameters were generally the same as recommended (Serin et al., 2021): 40-fold, 50 repeated cross-validation (CV) procedures, hyperparameters with the grid search algorithm, auto optimization for classification algorithms, two contrasts, a start value of *p* of 0.01 for the 68 × 68 matrix, and a start value of *p* is 0.005 for the 360 × 360 matrix.

### 2.8. White matter hyperintensity (WMH)

WMH burden was automatically measured using individual T1 anatomical and 3D T2-FLAIR images with the Lesion Segmentation Tool (LST^4^; Schmidt et al., 2012) as described in its tutorial and our recent publications (Gao et al., 2019, 2021a,b). WMH lesions were segmented by the lesion growth algorithm with a default initial threshold (*κ* = 0.3), and total WMH number and size (ml) were generated. The binary WMH segments were spatially normalized into the standard Montreal Neurological Institute (MNI152) space and summarized for group visualization.

### 2.9. Statistical analysis

For cognitive tests, age, and education, group statistics were carried out using SPSS 16.0 (SPSS Inc., Chicago, IL, United States) with a significance threshold of *p* < 0.05; for gender, diabetes, hyperlipidemia, and smoking, group statistics were determined using Chi-square tests with a significant *p* < 0.05. For eSC, a threshold for the false discovery rate (FDR) *p* < 0.05 was used to address multiple comparisons.

To understand the relationship between suprathreshold edge and network measures (i.e., eSC, system segregation, and integration scores), cognitive tests, and WMH burden, it is instructive to correlate these metrics either using univariate nonparametric correlation tests or NBS-based regressions. First, suprathreshold edges were used for the prediction of cognitive performance and WMH burden with the NBS-Predict toolbox to generate confidence intervals by repeating the CV procedure 50 times. Next, a nonparametric *Spearman* correlation analysis was conducted to examine associations between the system segregation and integration scores and neurobehavioral tests and WMH burden using SPSS 16.0 (SPSS Inc., Chicago, IL, United States) and GraphPad Prism 6 (GraphPad Software, Inc., La Jolla, United States).

## 3. Results

### 3.1. Demographic and clinical data

**Figure 2:**
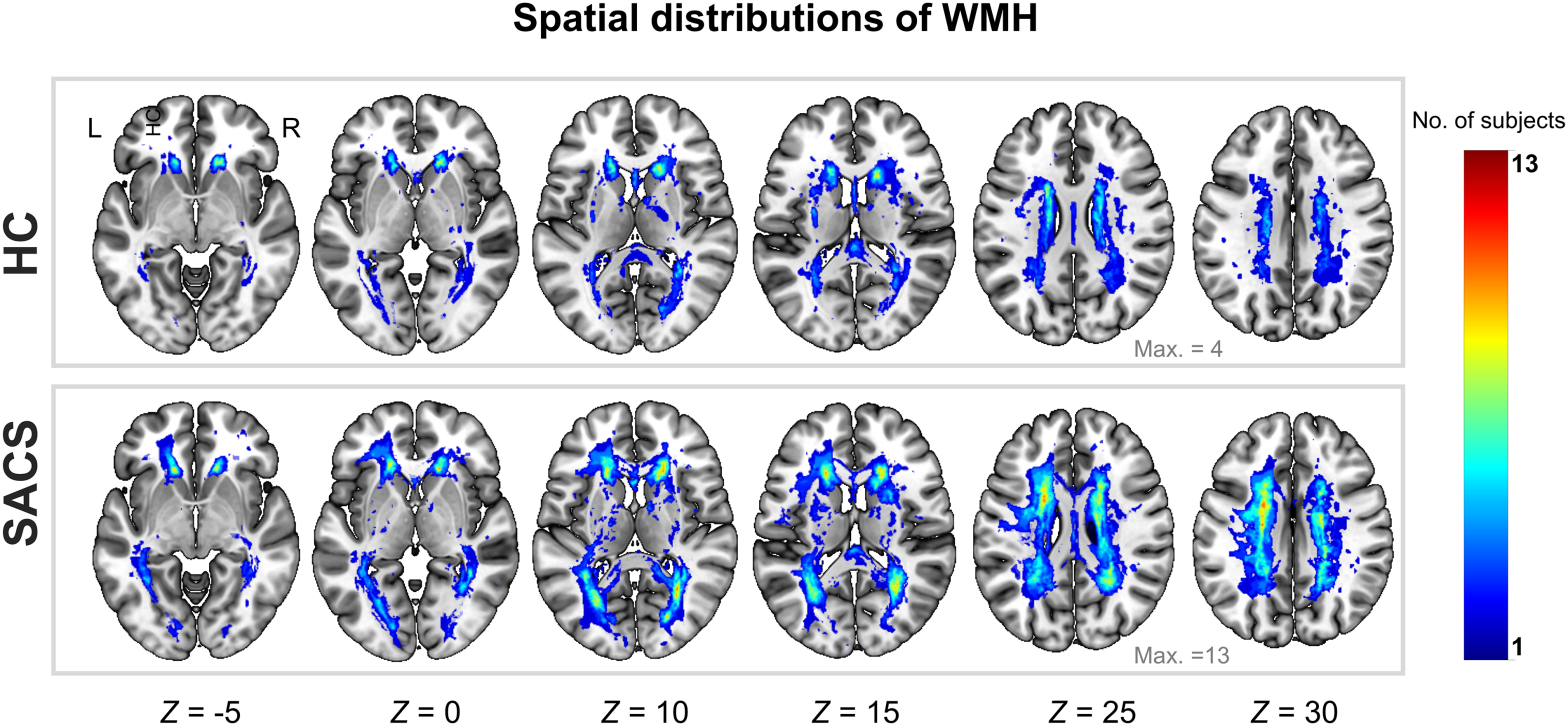
Probability map of WMH lesions. Spatial distributions of cumulative WMH for the control group (upper panel) and patients with SACS (lower panel). The colorbar indicates the number of subjects who had WMH in the same voxel, i.e., the control group had a maximum of 4 subjects overlapping in the same voxel, while the SACS patients had a maximum of 13 subjects overlapping in the same location. L, left; R, right; WMH, white matter hyperintensity; SACS, severe asymptomatic carotid stenosis; HC, healthy controls.

As shown in Table 1, the SACS patients had comparable demographics with the HC on gender, age, education, underlying diseases (hypertension, diabetes, hyperlipidemia), and smoking (*ps* > 0.05). However, they performed significantly worse on tests of verbal memory (both immediate and delayed recall memories, *ps* < 0.005), global cognition (MMSE and MoCA, *ps* < 0.05), executive functions (DST, *p* < 0.05), and higher WMH load (ratio between WMH size and total brain size, WMH volumes, and WMH number, *ps* < 0.005; Table 1 and Figure 2).

**Table 1:** Demographics and clinical characteristics.

	SACS	HC	*p*value
Age (years)	64.3 (7.2)	67.2 (6.1)	0.17
Sex			0.99^a^
Male	15	19	
Female	4	5	
Education (years)	9.6 (2.8)	11.1 (3.5)	0.12
Hypertension	17 (89%)	18 (75%)	0.23
Diabetes	4 (21%)	4 (17%)	0.71
Hyperlipidemia	9 (47%)	11 (46%)	0.92
Smoke	9 (47%)	6 (25%)	0.13
Affected side	7 l/12R	–	–
MMSE	26.8 (0.7)	27.4 (0.7)	0.015*
MoCA	23.3 (1.2)	24.2 (1.6)	0.017*
Word fluency	33.4 (6.2)	37.1 (4.0)	0.258
Digit Symbol Tests	28.0 (4.7)	31.5 (5.5)	0.029*
Backwards digit-span	5.8 (1.0)	6.5 (0.9)	0.042*
Forwards digit-span	3.8 (0.8)	4.5 (0.8)	0.021*
Immediate recall	31.0 (4.5)	35.8 (5.6)	0.004**
Delayed recall	4.6 (1.6)	6.5 (1.1)	<0.001***
WMH corrected	0.6 (0.7)	−0.6 (1.0)	<0.001***
WMH size (mL)	10.8 (3.5)	1.6 (3.1)	<0.005**
WMH number	13.4 (5.8)	5.9 (6.3)	<0.001***

Values are presented as mean (SD) or number (%). L, left; R, right; MMSE, Mini-Mental State Examination; MoCA, Montreal Cognitive Assessment; a, two-tailed Chi-squared test; WMH, white matter hyperintensity; WMH corrected, white matter hyperintensity (corrected by total cranial volume and log10 transformed); WMH size, white matter hyperintensity volumes; WMH number, number of WMH.* represents *p*< 0.05, ** *p*<0.01, and *** *p*<0.001

### 3.2. Group-average edge-centric structural connectivity and between-group differences in edge-centric structural connectivity

**Figure 3:**
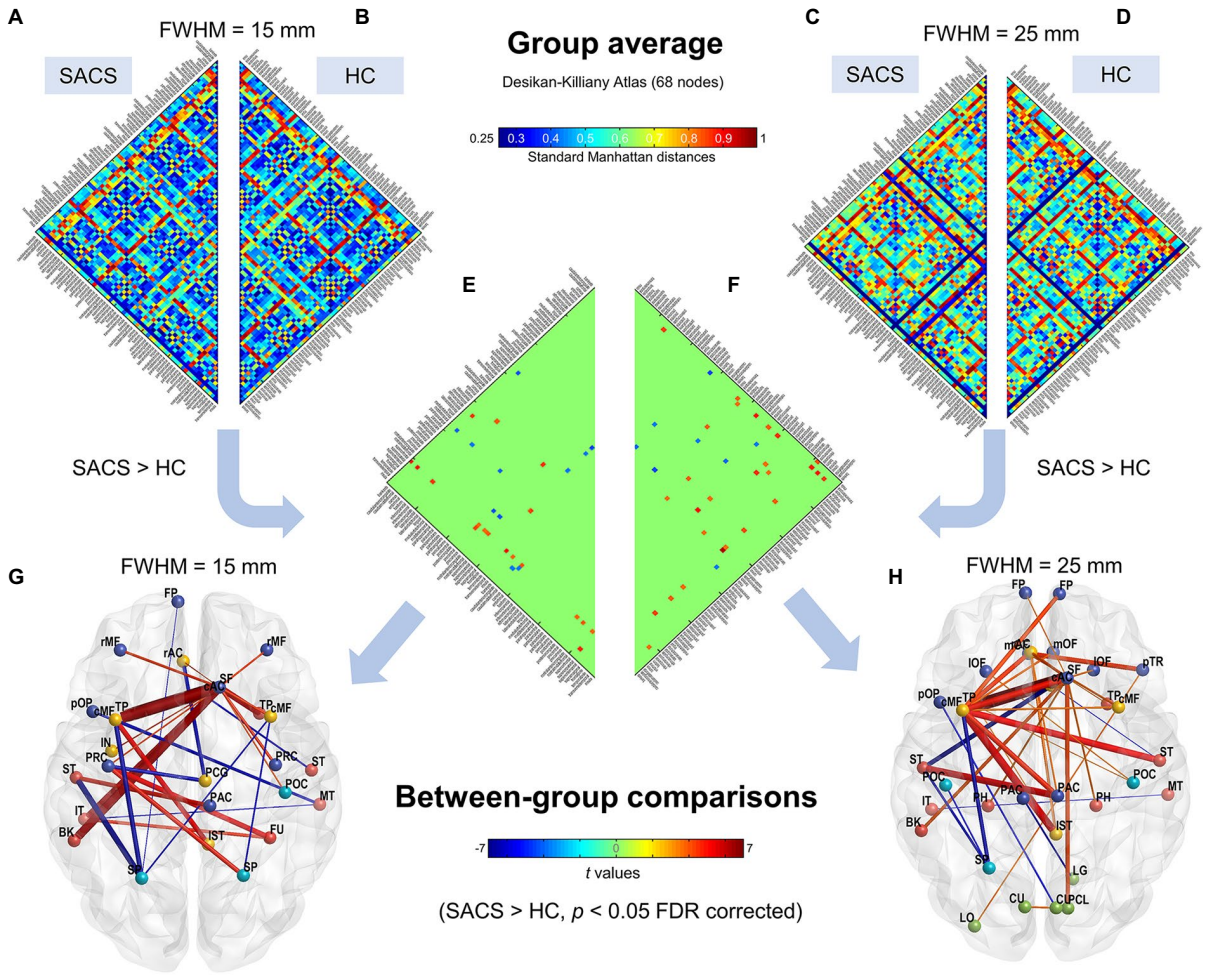
Subject-level network using the Desikan-Killiany atlas (68 parcels). Group average connectivity matrices for SACS patients **(A,C)** and healthy controls **(B,D)** with 15 mm and 25 mm smoothing kernels using the Desikan-Killiany atlas are shown. The colorbar shows standard Manhattan distances, with shorter values reflecting greater structural connectivity and vice versa. Subgraphs **(E,F)** show between-group differences on single-subject network connectivity with 15 mm and 25 mm smoothing kernels, respectively, which could be visualized as graphs. **(G,H)** The colorbar in the lower panel shows *t* values for the group statistics on edges, with a positive *t*-value representing that the SACS patients had a longer Manhattan distance and thus a lower structural connectivity on the edge, and vice versa. LH, left hemisphere; RH, right hemisphere.

Group-average eSC for the SACS and HC groups is shown in Figure 3. A long Manhattan distance reflects a weak morphological connection; in contrast, a short one reflects a short traveling distance or a strong connection.

We first examined eSC with the canonical Desikan-Killiany atlas. The Manhattan distances of the interregional CT histograms revealed significant changes in eSC in SACS patients. The SACS patients showed higher interhemispheric but shorter intrahemispheric Manhattan distances; in other words, they had decreased interhemispheric but increased intrahemispheric morphological connectivity seeded from left lateral temporal regions (Figure 3). They also had shorter distances between somatosensory/motor regions (Figure 3).

Using the finer HCP-MMP atlas, we further examined and validated the results. Generally, suprathreshold edges replicated those in the Desikan-Killiany atlas, however, the HCP-MMP atlas yielded additional significant edges. These unique edges included significantly shorter distances both in inter- and intra- hemispheric pairs seeding from the right lateral occipital regions (Figure 3).

### 3.3. Validation analysis

**Figure 4:**
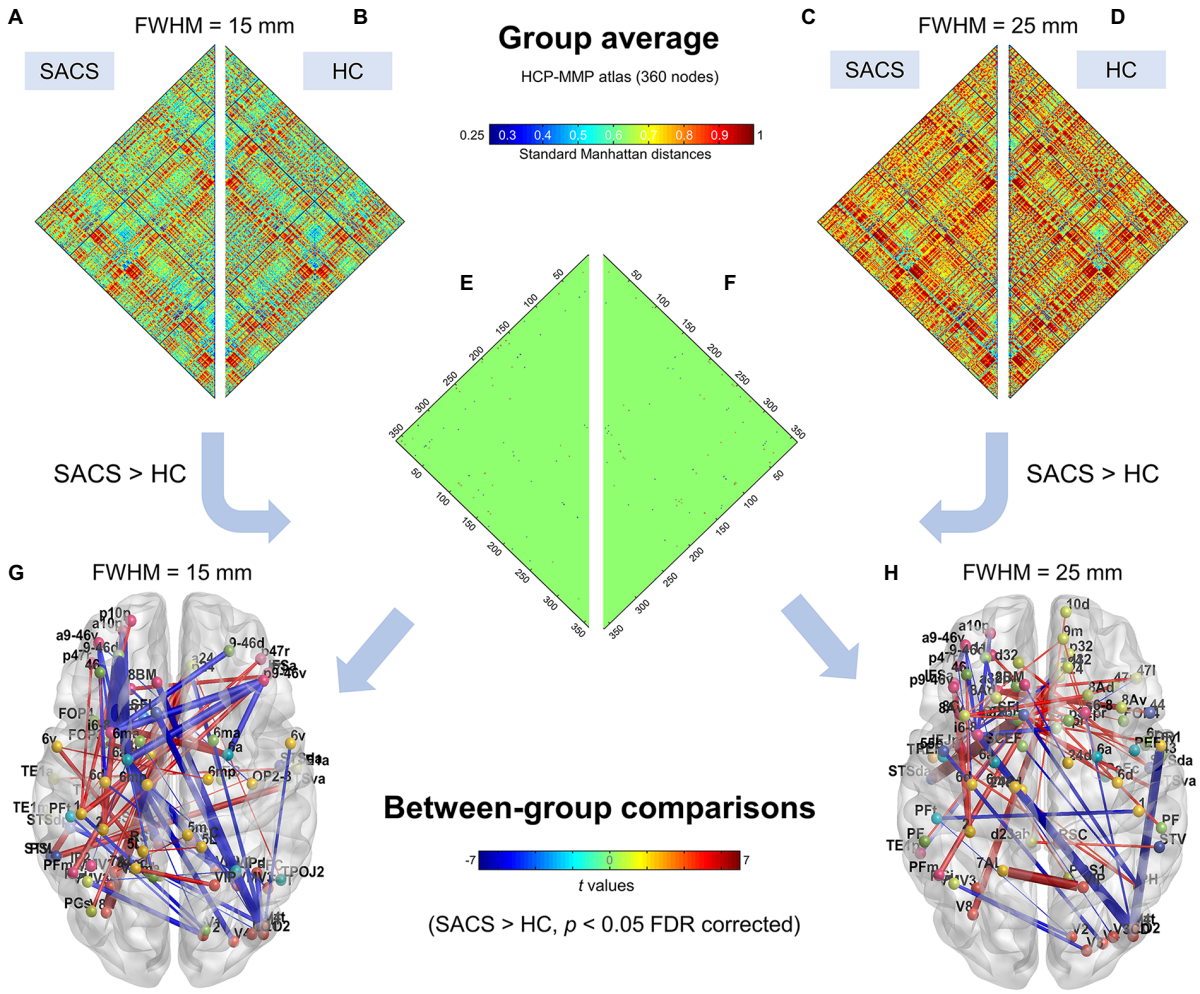
Individualized single-subject networks using the HCP-MMP atlas (360 parcels). Group average connectivity matrices for SACS patients **(A,C)** and healthy controls **(B,D)** with 15 mm and 25 mm smoothing kernels using the HCP-MMP atlas are shown. The colorbar shows standard Manhattan distances, with smaller values reflecting greater structural connectivity and vice versa. Subgraphs **(E,F)** show between-group differences on network connectivity with 15 mm and 25 mm smoothing kernels, respectively, which could be visualized as graphs. **(G,H)** The colorbar shows *t* values for the group statistics on the edges, with a positive *t*-value representing that the SACS patients had a longer Manhattan distance and thus a lower structural connectivity on the edge, and vice versa. LH, left hemisphere; RH, right hemisphere.

We further used a different spatial smoothing kernel, i.e., a 25-mm FWHM, to validate the group comparison results and found they were generally comparable (Figures 3, 4). A larger FWHM brought higher local similarity in CT values and, therefore, shorter Manhattan distances and a higher eSC.

### 3.4. Network-based statistic prediction

**Figure 5:**
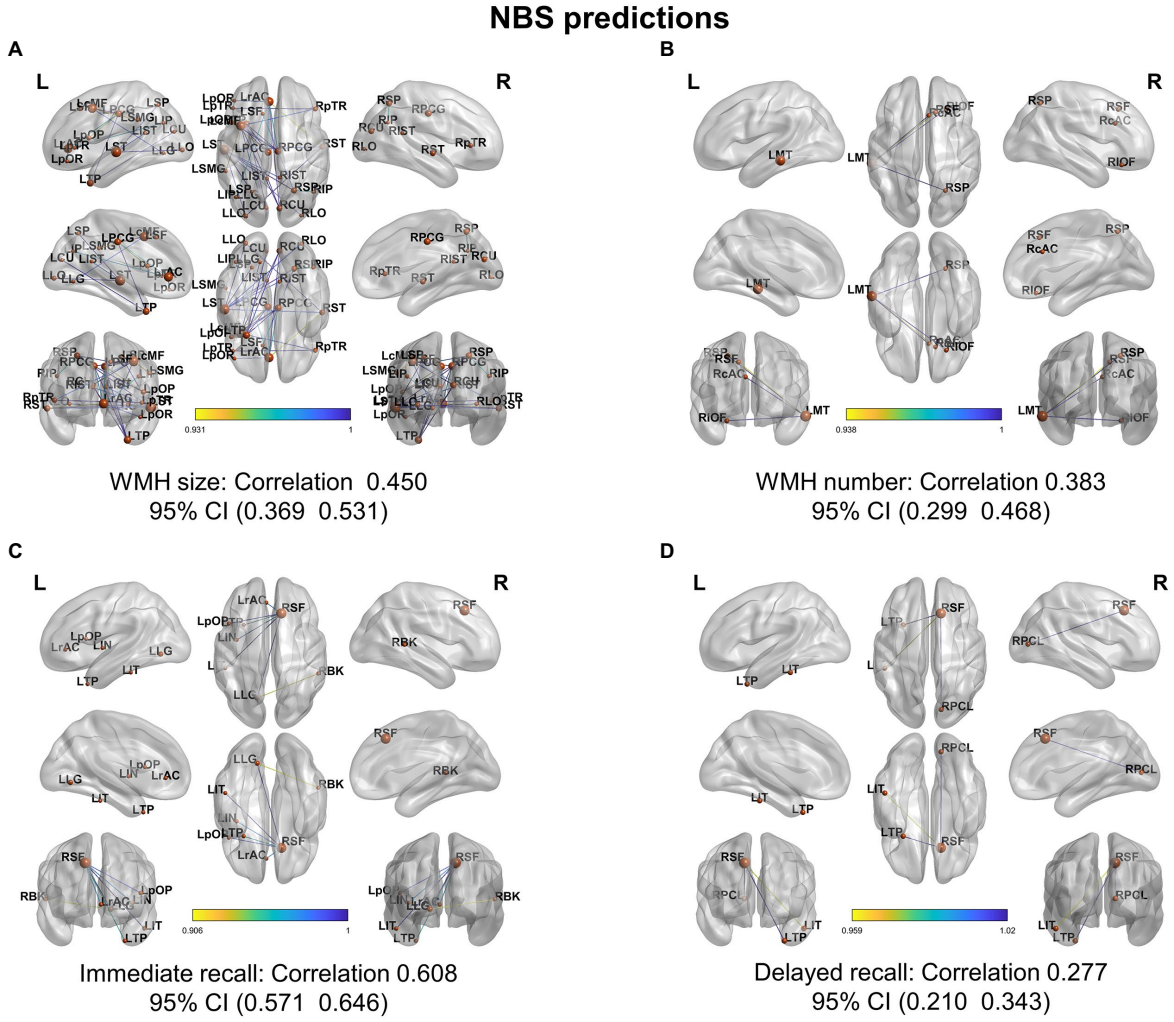
NBS classification. In patients with SACS, the NBS classification identifies both hyper-connected (upper panel) qqand hypo-connected (lower panel) subnetworks. Hyper-connected subnetworks (weight threshold = 0.9) in patients are depicted in **(A)** the suprathreshold adjacent matrix, **(B)** classification performance, **(C)** a graph visualized with the BrainNet Viewer (https://www.nitrc.org/projects/bnv/), and **(D)** the circular connectome. Edges in both figures, and nodes in the circular graphs are colored according to their weights and standardized nodal degree.

We investigated the biomarkers of SACS using the eSC data from the SACS patients and HC. Using NBS prediction, we identified both hypo- and hyper-connected subnetworks in patients with SACS, including brain regions located in the language, somatosensory/motor, frontoparietal, and salience systems (Figure 5 and Table 2).

**Table 2:** Nodes with reduced connections and their degree.

Nodes	Degree	Nodes	Degree	Nodes	Degree
r_superiorfrontal (RSF)	22	l_lateralorbitofrontal (LIOF)	2	l_pericalcarine (LPCL)	1
l_caudalmiddlefrontal (LcMF)	12	l_middletemporal (LMT)	2	l_precentral (LPRC)	1
r_isthmuscingulate (RIST)	8	l_parsopercularis (LpOP)	2	l_rostralmiddlefrontal (LrMF)	1
l_medialorbitofrontal (LmOF)	7	l_precuneus (LPCU)	2	l_insula (LIN)	1
l_rostralanteriorcingulate (LrAC)	4	l_superiortemporal (LST)	2	r_caudalanteriorcingulate (RcAC)	1
r_caudalmiddlefrontal (RcMF)	4	l_frontalpole (LFP)	2	r_lateraloccipital (RLO)	1
r_postcentral (RPOC)	4	r_lateralorbitofrontal (RIOF)	2	r_medialorbitofrontal (RmOF)	1
l_parsorbitalis (LpOR)	3	r_parstriangularis (RpTR)	2	r_parahippocampal (RPH)	1
l_temporalpole (LTP)	3	r_rostralanteriorcingulate (RrAC)	2	r_parsopercularis (RpOP)	1
r_bankssts (RBK)	3	r_temporalpole (RTP)	2	r_precuneus (RPCU)	1
r_paracentral (RPAC)	3	l_lateraloccipital (LLO)	1	r_rostralmiddlefrontal (RrMF)	1
r_pericalcarine (RPCL)	3	l_lingual (LLG)	1	r_superiorparietal (RSP)	1
l_bankssts (LBK)	2	l_parahippocampal (LPH)	1	r_superiortemporal (RST)	1
l_inferiortemporal (LIT)	2	l_parstriangularis (LpTR)	1	r_frontalpole (RFP)	1

### 3.5. System segregation and integration

**Figure 6:**
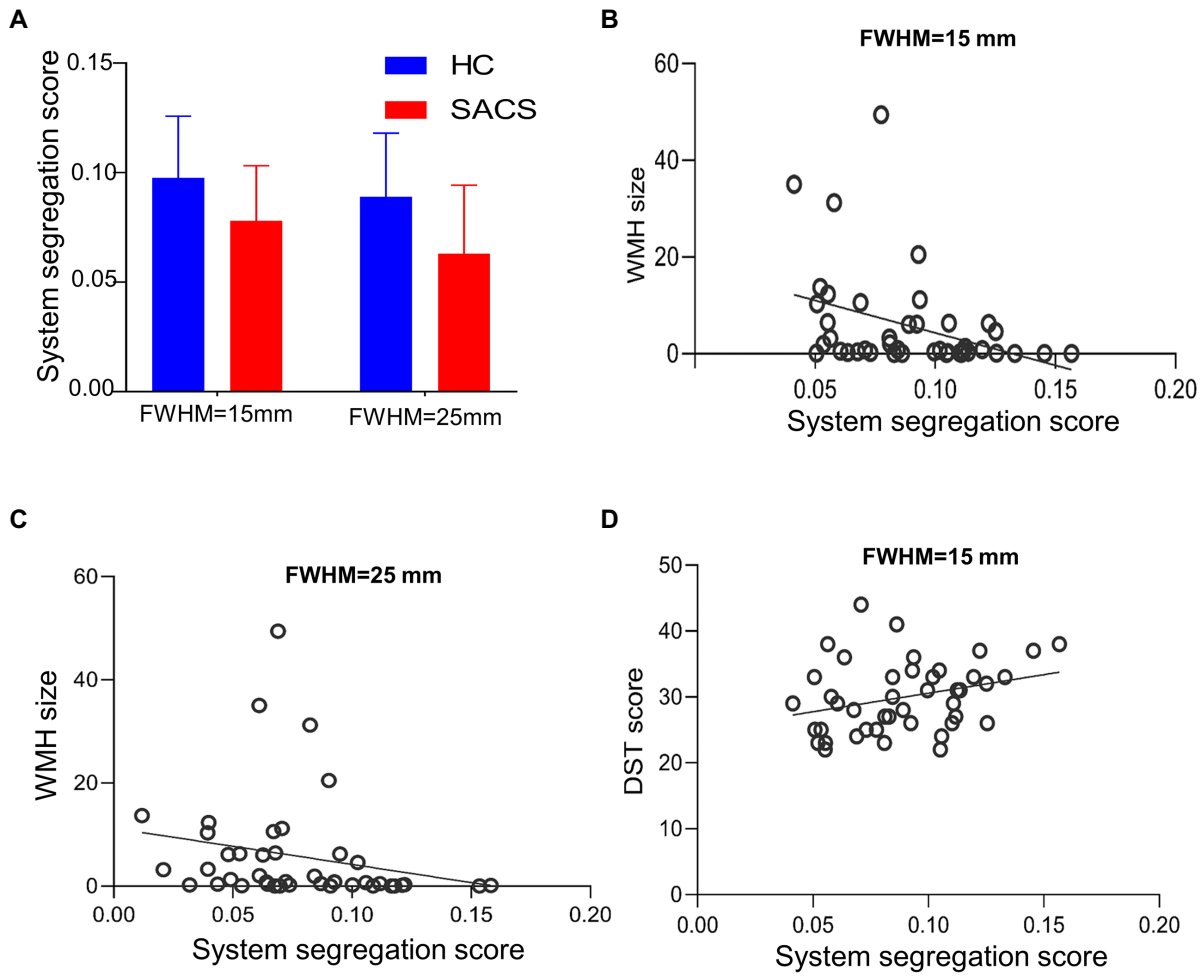
System segregation analysis. Between group comparisons on system segregation scores for the Desikan-Killiany atlas, both with FWMH 15 mm and 25 mm **(A–D)**. The SACS have significantly lower segregation scores, which are also correlated with the burden of white matter hyperintensity and cognitive tests.

The SACS patients had significantly lower system segregation (0.078 ± 0.025 *vs* 0.098 ± 0.028, *t* = − 2.411, *p* = 0.021) at 15-mm FWMH, and (0.063 ± 0.031 *vs* 0.089 ± 0.029, *t* = − 2.811, *p* = 0.008) at 25-mm FWMH (Figure 6A) using the Desikan-Killiany atlas. Using either size or number, a lower system segregation score was significantly associated with a higher WMH burden (Figures 6B,C). Conversely, a higher system segregation score was significantly associated with better DST scores (Figure 6D).

### 3.6. Network-based statistical prediction

**Figure 7:**
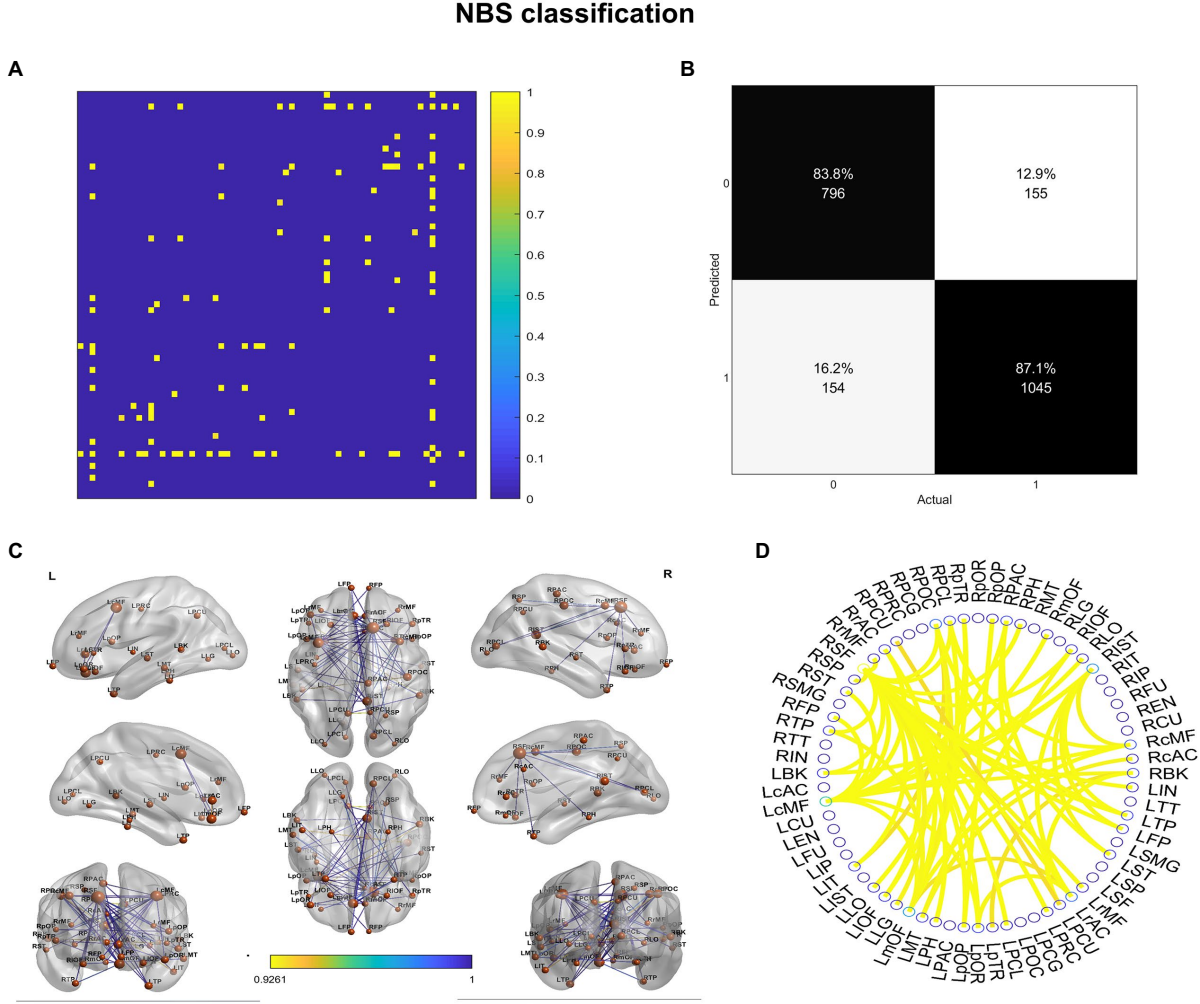
NBS-based regression on white matter hyperintensity and cognitive tests. NBS predicted WMH burden and cognitive performance from a structural network of interareal cortical thickness histograms with Pearson’s correlation coefficients (95% CI) between predicted and actual WMH burden and recall memory for **(A)** white matter hyperintensity (WMH) size, **(B)** white matter hyperintensity number, **(C)** immediate recall, and **(D)** delayed recall memories.

Further, NBS-predict regression analysis showed that structural hypoconnectivity (negative) predicted WHM burden with Pearson’s correlation coefficients of 0.450 (95% CI: 0.369, 0.531) in WMH size (Figure 7A) and of 0.383 (95% CI: 0.299, 0.468) in WMH number (Figure 7B), respectively. Comparatively, structural connectivity (positive) predicted cognitive tests with Pearson’s correlation coefficients of 0.608 (95% CI: 0.571, 0.646) in immediate recall (Figure 7C) and of 0.277 (95% CI: 0.210, 0.343) in delayed recall (Figure 7D), respectively.

## 4. Discussion

We estimated the Manhattan distances between pairwise CT histograms to compute eSC and construct subject-wise morphological brain networks. This analysis showed that the SACS patients had longer interhemispheric but shorter intrahemispheric Manhattan distances seeding from left lateral temporal regions, corresponding to lower interhemispheric but higher intrahemispheric connectivity. Further network analysis showed that the SACS patients had decreased system segregation paralleling with WMH burden and recall memory. Lastly, we applied a recently developed NBS approach to get feature weights for eSC, and subgraph features that predicted WMH load and cognitive tests. These results showed that SACS had disrupted structural connectivity, altered eSC measured by Manhattan distances, and pronounced lower system segregation. Further, NBS analysis yielded good feature classification performance. These findings suggest that subject-wise eSC has the potential to identify high-risk individuals while also providing insights into the morphological connectivity basis of cognitive impairment and accelerated aging in advanced asymptomatic carotid stenosis.

This study identified that patients with SACS exhibited altered pairwise CT co-vary patterns with increased interhemispheric but decreased intrahemispheric Manhattan distances seeding from the left lateral temporal regions. This result is consistent with prior reports on atrophy and dysconnectivity in the same regions (e.g., Cheng et al., 1970; Lin et al., 2014; Avelar et al., 2015; Marshall et al., 2017; Tani et al., 2018; Nickel et al., 2019; Gao et al., 2021b), and also generally consistent with previous reports using fMRI functional connectivity or diffusion tensor imaging SC (Lin et al., 2014; Huang et al., 2018; Gao et al., 2019; He et al., 2020). For example, earlier results from resting-state functional connectivity show that SACS has reduced interhemispheric connectivity, especially in the frontoparietal network ipsilateral to stenosis (Cheng et al., 1970). Our prior study also has identified lower interhemispheric functional connectivity located in the Perisylvian regions, spanning across somatomotor, salience, and dorsal attention networks (Gao et al., 2019). We interpret this finding as altered morphological co-variations dominated by the lateral temporal regions. Notably, the SACS exhibited shorter Manhattan distances and thus a higher eSC. This is unusual since neurodegenerative diseases are often associated with decreased connectivity globally. On the one hand, this phenomenon may reflect pairwise synchronized/co-vary atrophy patterns, as much of previous evidence has shown that these regions have thinner cortex in SACS (Avelar et al., 2015; Marshall et al., 2017; Nickel et al., 2019; Gao et al., 2021a), and this synchronized shrinkage could lead to an increased similarity. This speculation requires further verification through computational modeling. On the other hand, this may reflect compensation in the preclinical stage of the disease, as many neurodegenerative diseases have shown a compensatory increase in the early stages.

It is also possible that in SACS patients, the long-range connections that maintain interhemispheric interactions and support advanced cognitive function are impaired, while the relatively short-range intrahemispheric connections that are essential for functional specification are enhanced. Neuroimaging studies have established that interhemispheric integration and intrahemispheric specialization are important underpinnings of an individual’s higher cognitive abilities (e.g., Buckner and Krienen, 2013).

Another novel finding of our study is that the SACS patients exhibited decreased system segregation and comparable system integration as compared to the controls. Human functional imaging has shown that system segregation decreases with normal aging (Chan et al., 2014) and supports cognitive resilience in Alzheimer’s disease (Ewers et al., 2021), and this reduction can be improved through visual speed processing training (Chen et al., 2021). This suggests that the community structure of SACS blurs system and network boundaries, which may reflect a compensatory mechanism under the condition of chronic hypoperfusion and compromised hemodynamics. Such phenomenology has also been suggested as decreased within-system and increased between-system functional connectivity in mild cognitive impairment and Alzheimer’s disease (Vipin et al., 2018).

Limitations. First, as a small sample study, we thus repeated the analyses using different smoothing kernels and atlases to overcome the potential bias; future large-sample studies are needed to address this issue and promote deep learning-based prediction of high-risk individuals, with emphasis on dementia and stroke, to inform clinical decisions for surgical intervention in such patients. Second, since morphological networks and functional connectivity networks have apparently different community structures, the network segregation and integration based on their calculations show different patterns, which is also an active research direction. Third, longitudinal data can provide information on disease progression and prediction of high-risk individuals, together with other clinically commonly used imaging modalities for silent lesion detection, which can lead to better diagnosis and prognosis.

## 5. Conclusion

Our results show that SACS exhibits abnormal subject-wise structural connectivity and a less-optimized trade-off between physical cost and network segregation, suggesting asymmetric and/or synchronous cortical atrophy under potential chronic hypoperfusion and disordered hemodynamic pressure. These results also provide a reference and perspective for a future large sample identifying high-risk carotid stenosis individuals.

## Data availability statement

The original contributions presented in the study are included in the article/supplementary material, further inquiries can be directed to the corresponding authors.

## Ethics statement

The studies involving human participants were reviewed and approved by the Medical Ethics Committee of Zhongnan Hospital of Wuhan University. The patients/participants provided their written informed consent to participate in this study.

## Author contributions

JR and DX analyzed the data and drafted the manuscript. LG supervised the neuroimaging analyses. HM, XZ, and CF collected the data. JR, XZ, JL, and YX contributed to the data analyses and wrote the manuscript. LG and HX conceived the idea and contributed to the manuscript editing. All authors contributed to the article and approved the submitted version.

## Funding

This study was supported by the National Natural Science Foundation of China (Grant No. 82001799 to LG) and was partially supported by the Shenzhen Science and Technology Innovation Commission (JCYJ20200109144801736), National Social Science Foundation of China (Nos. 20 & ZD058), and National Natural Science Foundation of China (No. 72031009).

## Conflict of interest

The authors declare that the research was conducted in the absence of any commercial or financial relationships that could be construed as a potential conflict of interest.

## Publisher’s note

All claims expressed in this article are solely those of the authors and do not necessarily represent those of their affiliated organizations, or those of the publisher, the editors and the reviewers. Any product that may be evaluated in this article, or claim that may be made by its manufacturer, is not guaranteed or endorsed by the publisher.

## Footnotes

^1^ http://surfer.nmr.mgh.harvard.edu/

^2^ https://github.com/raamana/graynet

^3^ https://www.nitrc.org/projects/nbspredict

^4^ www.applied-statistics.de/lst
